# The treatable intellectual disability APP www.treatable-id.org: A digital tool to enhance diagnosis & care for rare diseases

**DOI:** 10.1186/1750-1172-7-47

**Published:** 2012-07-23

**Authors:** Clara D M van Karnebeek, Roderick F A Houben, Mirafe Lafek, Wynona Giannasi, Sylvia Stockler

**Affiliations:** 1 Division of Biochemical Diseases, Department of Pediatrics, BC Children’s Hospital, University of British Columbia, Vancouver, Canada; 2 Treatable Intellectual Disability Endeavor in British Columbia (TIDE-BC), Vancouver, Canada; 3 Health2Media, Vancouver, Canada; 4 Howegroup Public Sector Consultants Inc., Vancouver, Canada; 5 Division of Biochemical Diseases, Department of Pediatrics, B.C. Children’s Hospital, Rm K3-201 4480 Oak Street, Vancouver, BC, V6H 3V4, Canada

**Keywords:** Inborn errors of metabolism, intellectual disability, treatment, knowledge translation, APP, digital tool, information portal

## Abstract

**Background:**

Intellectual disability (ID) is a devastating and frequent condition, affecting 2-3% of the population worldwide. Early recognition of treatable underlying conditions drastically improves health outcomes and decreases burdens to patients, families and society. Our systematic literature review identified 81 such inborn errors of metabolism, which present with ID as a prominent feature and are amenable to causal therapy. The WebAPP translates this knowledge of rare diseases into a diagnostic tool and information portal.

**Methods & results:**

Freely available as a WebAPP via http://www.treatable-id.org and end 2012 via the APP store, this diagnostic tool is designed for all specialists evaluating children with global delay / ID and laboratory scientists. Information on the 81 diseases is presented in different ways with search functions: 15 biochemical categories, neurologic and non-neurologic signs & symptoms, diagnostic investigations (metabolic screening tests in blood and urine identify 65% of all IEM), therapies & effects on primary (IQ/developmental quotient) and secondary outcomes, and available evidence For each rare condition a ‘disease page’ serves as an information portal with online access to specific genetics, biochemistry, phenotype, diagnostic tests and therapeutic options. As new knowledge and evidence is gained from expert input and PubMed searches this tool will be continually updated. The WebAPP is an integral part of a protocol prioritizing treatability in the work-up of every child with global delay / ID. A 3-year funded study will enable an evaluation of its effectiveness.

**Conclusions:**

For rare diseases, a field for which financial and scientific resources are particularly scarce, knowledge translation challenges are abundant. With this WebAPP technology is capitalized to raise awareness for rare treatable diseases and their common presenting clinical feature of ID, with the potential to improve health outcomes. This innovative digital tool is designed to motivate health care providers to search actively for treatable causes of ID, and support an evidence-based approach to rare metabolic diseases. In our current –omics world with continuous information flow, the effective synthesis of data into accessible, clinical knowledge has become ever more essential to bridge the gap between research and care.

## Background

*Intellectual disability (ID)* is a life-long and debilitating condition with deficits in cognitive functioning (IQ < 70) and adaptive skills [1,2]. ID is often associated with behavioural problems (autism, hyperactivity, aggressivity and self-injurious behaviour), epilepsy and other neurological disabilities, all resulting in psychological, social and economic burdens [3,4]. In children <5 yrs of age with deficits in two or more developmental domains (e.g. fine/gross motor skills, speech, interaction, etc.), the term global developmental delay (DD) is applied [5]. Here we will use the term ID collectively for both ID and DD. ID is frequent, affecting 2-3% of children and adults worldwide and is the disease category with one of the largest health care costs [6]. The etiology of ID is diverse, including infectious, traumatic and toxic causes. Genetic etiologies constitute the most frequent cause and are demonstrable in more than 50% of individuals with ID [7], ranging from numeric and structural chromosomal abnormalities and submicroscopic Copy Number Variants to methylation abnormalities, and to single gene defects [8].

Current guidelines aimed at structuring the evaluation of genetic causes of ID are based on frequencies of single conditions and yield of diagnostic methods and procedures [9]. Therefore, karyotyping and array-comparative genomic hybridisation, which yield a causal diagnosis in 20% of cases, is standard practice as part of the first-line investigation [10,11]. Unfortunately these high diagnostic yields do not translate into therapeutic benefit, as at the present time, causal therapy is not available for most conditions identified by these investigations. One category of genetic conditions is amenable to treatment however: inborn errors of metabolism (IEM).

However, because the single conditions are rare (e.g. Phenylketonuria 1:10.000) to ultrarare (e.g. Guanidinoacetate methyltransferase (GAMT) deficiency 1:200.000) and diagnosis is considered complicated and expensive, they are not systematically screened in a child with ID [12]. Expanded newborn screening covers some but by far not all of them, and may miss mild forms of disease.

In order to assess the number of currently treatable IDs we recently performed a systematic literature review, and identified 81 treatable IEM with ID as a major clinical feature [13]. While 60% of these conditions can be detected through a panel of widely available screening tests on blood and urine (e.g. aminoacids, homocysteine, copper, ceruloplasmin, organic acids, purines & pyrimidines, creatine & guanidinoacteate, glycosaminoglycans & oligosaccharides), for the remaining 35% conditions (n = 28) a ‘single test per single disease’ APProach including single metabolite or primary molecular analysis is required. Because these tests may be difficult to obtain, and / or require extensive funding, and / or require invasive sampling procedures (spinal tap for cerebrospinal fluid collection, skin biopsy to cultivate fibroblasts), a clinical differential diagnosis is needed to provide efficiency in the diagnostic work up.

To mitigate the complexity and time-consuming nature of this task we have created a downloadable WebAPP http://www.treatable-id.org with the aim of facilitating the recognition of treatable ID and maximizing the efficiency of diagnostic work up. Providing an interactive tool for both clinicians and scientists this tool is intended to help to increase the general awareness of treatable ID and to create a reliable information portal for rare metabolic diseases.

## Methods & results

For the detailed methodology with results of our systematic literature review, the reader is referred to: *Molecular Genetics and Metabolism 2012 Mar;105(3):368–8*1, in pdf version freely downloadable via: http://www.sciencedirect.com/science/article/pii/S1096719211006081

### Parameters

We designed the digital APPlication for a *target audience* including all specialists evaluating children with ID (general and developmental pediatricians, neurologists, geneticists, metabolic specialists) as well as laboratory scientists, ranging from student to expert level.

We created menus showing the conditions according to biochemical categories, clinical signs & symptoms, diagnostic tests as well as therapies and evidence. We created a *disease page* for each of the 81 treatable IDs including providing a detailed information portal with information on all aspects of the particular rare disease with links to internationally accepted resources.

### Technology

The WebAPP was created using the latest web standards and is best viewed in the latest version of all major browsers (Explorer 8+, Safari, Chrome & Firefox). Furthermore the APP is designed such that it is easily accessible on all major tablets, e.g. the Apple iPpad. This whole process was supported and funded by the ‘Metakids Foundation’ in The Netherlands (http://www.metakids.nl).

The Digital Tool is freely available as a WebAPP via http://www.treatable-id.org. Users are requested to register online. In the middle of 2012 this tool will also be downloadable via the iOS & Android APP stores for use on mobile devices.

The APP will be updated (with novel data on diseases, diagnostics, treatments, evidence) at 3 month-intervals by performing predesigned searches in PubMed and selections according to previously described strategies. Users are asked for feedback and input via email and international experts will be asked to update and maintain particular disease pages (see below) which will be incorporated for continuous improvement.

## Design & use

**Figure 1:**
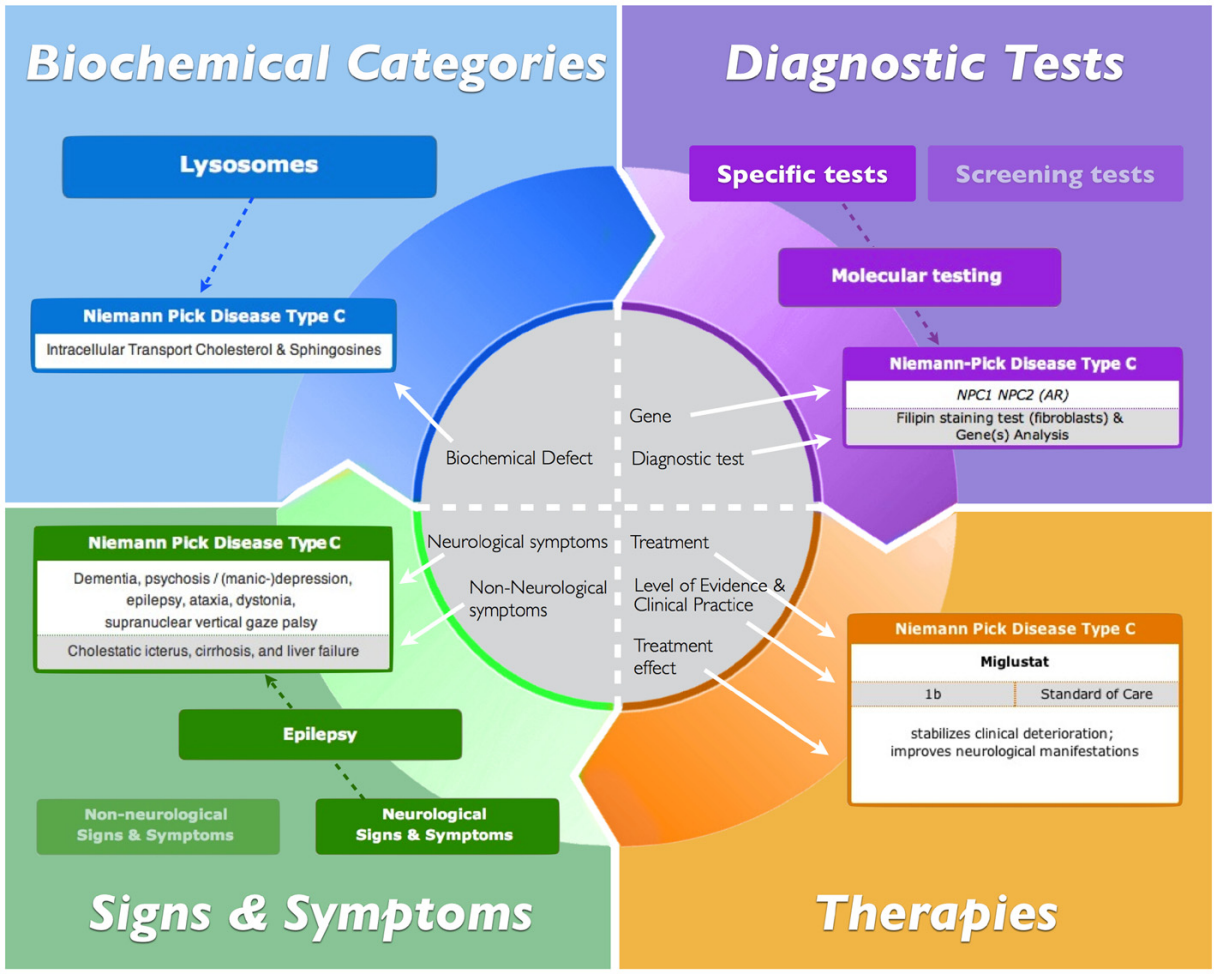
**WebAPP as knowledge translation tool to enhance diagnosis and treatment of intellectual disabilities.** The interactive menus include: biochemical group, signs & symptoms, diagnostic tests, treatment & evidence.

The collective information on the diseases, causally related to ID and amenable to treatment, is presented in several different ways as shown in Figure 1.

I) *Biochemical Categories*

The treatable diseases are presented in 15 biochemical categories according to accepted nomenclature and/or pathophysiology. For each disease the biochemical defect is listed, with illustration thereof provided on the ‘disease page’.

II) *Neurologic and Non-Neurologic Signs & Symptoms*

The clinical features for all rare diseases are divided into neurological and non-neurological signs and symptoms. For each rare disease only the most characteristic, specific and consistent features are listed.

*Neurologic features* include ataxia, behavioural disturbance, dementia, dystonia, encephalopathic crisis, epilepsy, hearing loss, hypotonia/myopathy, neuro-imaging abnormalities (basal ganglia, cerebellum, cerebrum, cysts/dysgenesis, white matter, mixed), neuropathy, ocular movement abnormality, psychiatric disturbance, sensorineural hearing loss, spasticity, stroke, vision loss. All IEM except one (Tyrosinemia type II) are associated with at least one additional prominent neurologic feature, of which the most frequent are epilepsy and various types and degrees of movement disorders (e.g. spasticity, dyskinesia, ataxia, etc). However, many of these conditions can present with ID as sole feature for a considerable time prior to manifestation of the full phenotype. A limitation of the list is the fact that in most case reports and series, the clinical presentation of the epileptic symptomatology and behavioural/psychiatric manifestations of IEM is poorly described. None are pathognomonic for a particular treatable ID. Due to this lack of knowledge, it is currently not possible to provide more detail on these particular signs and symptoms.

*The non-neurologic features* affect the following anatomic / organ systems: bones and joints, dermatology, endocrinology, eye, facial dysmorphism, growth & stature, heart, gastrointestinal, haematology, immunology, kidney, liver, odour. For 69% of the treatable IEM, a non-neurologic feature is a prominent part of the phenotype.

*In general,* it is emphasized that that absence or presence of specific signs and / or symptoms not fitting the list does not rule out the specific disorder in a patient. Also, these data are subject to change as new diagnostic techniques provide novel insights into the spectrum of phenotypic presentation and natural history of metabolic diseases, and will be updated accordingly.

It is also possible to search for a specific combination of signs and symptoms. This feature, highly valued by physicians in a baseline user survey, uses a search engine and displays the disease pages on this site that contain the signs and symptoms entered. This feature will be continuously improved based on user feedback and search input.

Finally, to further support the clinician in narrowing down the differential diagnosis, the APP displays a dichotomy: those identifiable by routine metabolic screening tests (white background) versus those requiring a specific test (green background). Thus the physician can immediately discard the ‘IEM with white background from the differential’ if routine metabolic screening was negative.

III) *Diagnostic Tests*

To facilitate a practical guide for biochemical and genetic diagnosis, the tests required for the diagnosis of each of the conditions were assessed. Accordingly, diseases were categorized into those diagnosed via ‘metabolic screening tests’ versus those diagnosed via a ‘single test per single disease’ approach.

*Screening Tests* were defined as those tests in blood and urine, which are readily available in biochemical laboratories in most developed countries, and with a yield of at least 2 IEM (and up to 22) per test, such as: plasma amino-acids and total homocysteine, copper, ceruloplasmin, urine organic acids, oligosaccharides, glycosaminoglycans, purines/pyrimidines, creatine metabolites (acylcarnitine profile may support these diagnosis but does not independently identify one of these IEM). Overall, these screening tests reliably provide clues for diagnosis for 65% of all treatable IDs.

For the remaining treatable conditions, a specific ‘*one test per one disease*’ approach is required. These diseases are listed accordingly under *Specific Tests* (including urine oligosaccharides and glycosaminoglycans)*.* At the time of publication of our review in 2012, primary gene analysis is the most reliable approach for 13 IEM (20 genes). Each disease button lists the causal gene(s) as well as the diagnostic test required.

*In general,* for most of the 81 diseases further confirmatory (biochemical / genetic) testing is needed for a definitive diagnosis.

IV) *Therapies*

The diseases are listed in alphabetical order with the following information for each: therapeutic modality/-ies, (ranging from supplements, diets, substrate inhibition to stem cell transplantation), level of evidence (ranging from 1 and 2 (20%) to 4–5 (majority), clinical practice (standard of care versus on an individual basis), effect on predefined primary (IQ/developmental quotient) and secondary (epilepsy, behavioural/psychiatric disturbances etc) outcomes.

V) *Disease Page*

**Figure 2:**
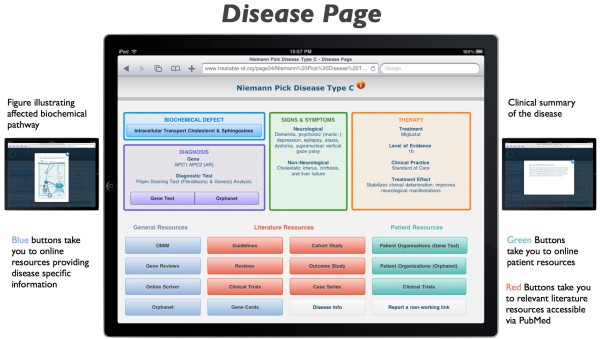
‘**Disease Page’ as information portal for each rare inborn error of metabolism.** As an example, the page with specific features and online resources for Niemann Pick Disease Type C is presented here. Levels of Evidence are defined as follows: (source: Centre for Evidence-Based Medicine, Oxford UK, www.cebm.net): Level 1a = Systematic Review of Randomized Controlled Trials (RCT), 1b = Individual RCT, 1c = ‘All or None’ (=(prolongation of) survival with therapy); Level 2a = Systematic Review of Cohort Studies, 2b = Individual Cohort Study, 2c = ‘Outcomes Research’ (focused on end results of therapy for chronic conditions, including functioning and quality of life (http://www.ahrq.gov/clinic.outfact.htm)); Level 3 = Systematic Review of Case–control Studies; Level 4 = Individual Case–control Study or Case-series/report; Level 4-5 = Single Case Report; Level 5 = Expert opinion without critical appraisal.

As illustrated by Figure 2, for each condition a ‘Disease Page’ has been designed as an information portal comprising an overview of all signs and symptoms, a figure showing the effected biochemical pathway, information on available diagnostic tests and causal therapies. In addition each page contains numerous online resources, including Orphanet, OMIM, Gene Reviews, Online Scriver, Gene Cards, journal articles, clinical trials, and patient resource websites.

## Evaluation and knowledge translation

Before launching the APP in our institution, the BC Children’s Hospital, a baseline survey, designed by an independent evaluator, was conducted with attending staff and trainees from Medical Genetics, Developmental Pediatrics, Neurology, Biochemical Genetics / Metabolic Diseases, Child Psychiatry (N = 15) to determine current practice and experience in the diagnostic evaluation of (treatable) ID. Findings indicated the volume of patients (mean: 8 per month), time spent searching for a diagnosis (mean: 3 hours, but at times exceeding 6 hrs), time spent confirming a diagnosis (mean: 11 months), and the proportion of causal diagnoses established (just over a quarter (28%) of cases). This data forms the baseline and will serve as a reference point for the diagnostic evaluation of ID prior to the APP.

Three focus groups were conducted with the same audience with the intent to determine clinicians’ initial feedback on the usability of the APP as well as comfort level with diagnosing and managing treatable IDs [14]. Via semi-structured interviews, users also were asked for their perceptions on functionality of the APP as well as suggestions for improving the uptake and usage of the APP [15]. Based on user feedback we integrated the following suggestions to optimize the current version of the APP: 1) search functions by signs and symptoms to help formulate a differential diagnosis; 2) possibility to save differential diagnoses for later comparison; 3) access via APP to trusted resources such as the most commonly used bibliographic databases (i.e. PubMed / Medline / Ovid Medline) and disease specific databases and on-line resources (i.e. OMIM, Gene Reviews, Scriver/OMMBID).

To enhance knowledge translation we have partnered with Child Health BC, a network of agencies working to build an integrated and accessible system of care for children and youth in the province of British Columbia, for the provision of capacity to support province-wide education and knowledge translation among General Practitioners, community pediatricians and specialists. These 3 groups of physicians will work collaboratively to achieve consensus on 1st tier metabolic screening for treatable IEM in all ID patients, communication pathways and appropriate referral to a tertiary care centre for further evaluation or treatment.

## Conclusions

### Being mindful of the gap

Despite continuous efforts to transform new insights generated by medical research into evidence-based clinical practice, this has proven difficult and has seldom translated into improved health outcomes [16,17]. For rare diseases, a field for which financial and scientific resources are particularly scarce, this gap is even more pronounced. Inherent to rare diseases the following challenges present itself: How can knowledge translation and dissemination be improved in a field in which: a) biological pathways are complicated and numerous; b) patients, as well as the physicians managing and scientists studying their diseases, are small in number, and internationally dispersed; c) clinical trials are few and far between; and hence d) evidence is limited?

### Time is Brain

New digital and social media, with the endless capacity to centralize information and connect people, provides an exciting new solution to these issues (e.g. Orphanet). With this APP we capitalize on technology to raise awareness for these rare treatable diseases, their common presenting clinical feature of ID, as well as the need for early diagnosis (‘Time is Brain’) to directly improve health outcomes.

This innovative digital tool is designed to motivate health care providers to search actively for treatable causes of ID, and support an evidence-based approach to rare metabolic diseases. In our current –omics world with continuous information flow, effective synthesis of data into accessible, clinical knowledge has become ever more essential to bridge the gap between research and care [18].

### Current applications

This APP was designed as part of our Treatable Intellectual Disability Endeavour (TIDE-BC; http://www.tidebc.org) research and care project. This funded project aims to improve health outcomes of all children with ID in the province of British Columbia, Vancouver through improved diagnosis and treatment. The APP is used by specialists in our institution as an essential part of our TIDE protocol, which was designed in consensus with international experts and superimposes the 1st and 2nd tier testing for treatable IDs to current international guidelines [8,11].

### Evaluation & ongoing improvement

To continuously improve and evaluate the impact of the tool, a mixed methodology evaluation will be conducted amongst online users and a local focus group, utilizing both formative and summative approaches. [15] Primary care physicians and specialists will be asked to provide their feedback on the utility of the APP in supporting the diagnostic evaluation. After registration online users are requested to provide feedback on the usefulness and applicability of the tool in their daily practice to support diagnosis and treatment of children with ID via the online APP feedback form. User visits to the site will also be tracked focusing on page usage (and non usage) and key search terminology (including signs and symptoms). The APP will be updated (with novel data on diseases, diagnostics, treatments, evidence) at 3 month-intervals, and improved through incorporation of data generated by our evaluation activities.

### Towards empowerment & better health outcomes

In the future this APP may be converted into an interactive information portal for patients and families, especially as new digital and social media (Twitter, blogs etc.) offer novel approaches to reaching and uniting rare disease patients from across the globe. Proven avid web users, patients / families in the rare diseases community may ultimately utilize new media as a vehicle for empowerment and to enable and better health outcomes. By increasing access to a larger volume of patients for clinical trials and increasing relevance of health outcomes studied and improved evaluation thereof, current research disseminated using innovative technologies will be effectively used to drive patient care improvements forward.

It is the hope that ‘one louder voice’ will support policymakers to make evidence based decisions that will result in the allocation of financial, scientific, and care resources to the rare diseases community.

## Abbreviations

(ID), intellectual disability; (DD), global developmental delay; (IEM), inborn errors of metabolism; (GAMT), guanidinoacetate methyltransferase.

## Competing interests

The authors declare that they have no competing interests.

## Authors’ contributions

CvK led the knowledge translation process from systematic literature review into this digital tool; she wrote and finalized this article. Funding from the BC Children’s Hospital Foundation first Collaborative Area of Innovation is acknowledged for CvK’s position as clinician-scientist in TIDE-BC. RH designed and programmed the treatable-ID APP and created the figures for this article. His work was funded by the Meta Kids Foundation in the Netherlands. (www.metakids.nl). ML coordinated and structured the search for and collection of data and online resources for the APP. WG performed and interpreted the usability testing, designed the framework for ongoing evaluation of the APP, and wrote and proofread this manuscript with focus on the evaluation section of the manuscript. SS co-authored this article and provided direction into the associated knowledge translation process presented here. SS is project leader of TIDE-BC.

## Authors’ information

CvK is the lead-clinician scientist of the TIDE-BC project, with a particular interest in diagnosis and treatment of rare diseases. She works as paediatrician specialized in metabolic diseases in BC Children’s Hospital, Vancouver. RH is founder and CEO of Health2Media (www.Health2Media.com; he specializes in the creation of online tools to enhance knowledge translation for physicians and scientists, and these activities for TIDE-BC. ML holds an M.D. diploma and is the lead research coordinator for TIDE-BC. WG is a founding partner of the Howegroup and has a vast experience in organisation and evaluation of health care projects. She is currently is the project manager for TIDE-BC. SS is an expert researcher in the field of neurometabolic diseases, head of the Biochemical Diseases Division in BC Children’s Hospital, as well as project leader for TIDE-BC.

## Funding

Stichting Metakids, Utrecht, The Netherlands (www.metakids.nl)

Rare Disease Foundation Vancouver, Vancouver, Canada (www.rarediseasefoundation.org)

‘1st Collaborative Area of Innovation’, BC Children’s Hospital Foundation, Vancouver Canada (www.tidebc.org)
